# Primary Intramedullary Spinal Melanoma: A Rare Disease of the Spinal Cord

**DOI:** 10.7759/cureus.16194

**Published:** 2021-07-05

**Authors:** Fatima Tuz Zahra, Zainub Ajmal, Jiang Qian, Stephen Wrzesinski

**Affiliations:** 1 Internal Medicine, Albany Medical Center, Albany, USA; 2 Pathology, Albany Medical Center, Albany, USA; 3 Hematology and Medical Oncology, Albany Medical Center, Albany, USA

**Keywords:** primary spinal melanoma, non cutaneous malignant melanoma, adjuvant radiation therapy, cancer-immunotherapy, rare brain tumors, neuro oncology

## Abstract

Primary malignant melanoma of the intramedullary region of the spinal cord has rarely been reported in the literature. These tumors can have variable appearance on magnetic resonance imaging (MRI) due to different extents of melanin and hemorrhage. Histopathologic confirmation and a comprehensive workup to rule out extra-spinal melanoma are required to make definitive diagnosis. We present a case of a patient diagnosed with primary intramedullary spinal melanoma in his lower thoracic spinal cord who was effectively treated with surgical resection, adjuvant radiation, and adjuvant immunotherapy. Gross total resection (GTR) is most vital in the management of this spinal tumor. Although several studies have established the efficacy of immunotherapy agents in advanced malignant melanoma, the use of these agents has not been studied in primary central nervous system melanomas. This case provides insight into the diagnostic approach and treatment options for this unique malignancy.

## Introduction

Primary melanoma of the central nervous system (CNS) is a rare neoplasm that accounts for approximately 1% of all cases of melanoma [1]. Primary intramedullary spinal cord melanoma is an extremely unique entity [2]. Diagnosis requires histopathological confirmation and ruling out metastatic spread from skin, ocular, or gastrointestinal primary lesions [3]. Surgical resection is the mainstay of treatment but due to rarity of the tumor, there are no well-defined guidelines for using available adjuvant therapies [2]. We present a case of primary intramedullary spinal melanoma treated with gross total resection and adjuvant radiation therapy followed by adjuvant immunotherapy with Nivolumab yielding favorable outcome.

## Case presentation

A 61-year-old male with no significant past medical history was seen by Neurology as an outpatient for evaluation of progressive lower extremity weakness, numbness and paresthesia, present for one year. He also complained of saddle paresthesia associated with urinary retention and stool incontinence. These symptoms had progressively worsened over several months with decreased lower extremity strength resulting in multiple falls.

On physical examination, the patient had asymmetric motor weakness in his bilateral lower extremities, worse on the right side. Complete loss of right hip abduction and right-sided foot drop was also noted. The patient had hypesthesia to temperature, proprioception, and vibration below the T11 sensory level. Deep tendon reflexes were normal.

**Figure 1:**
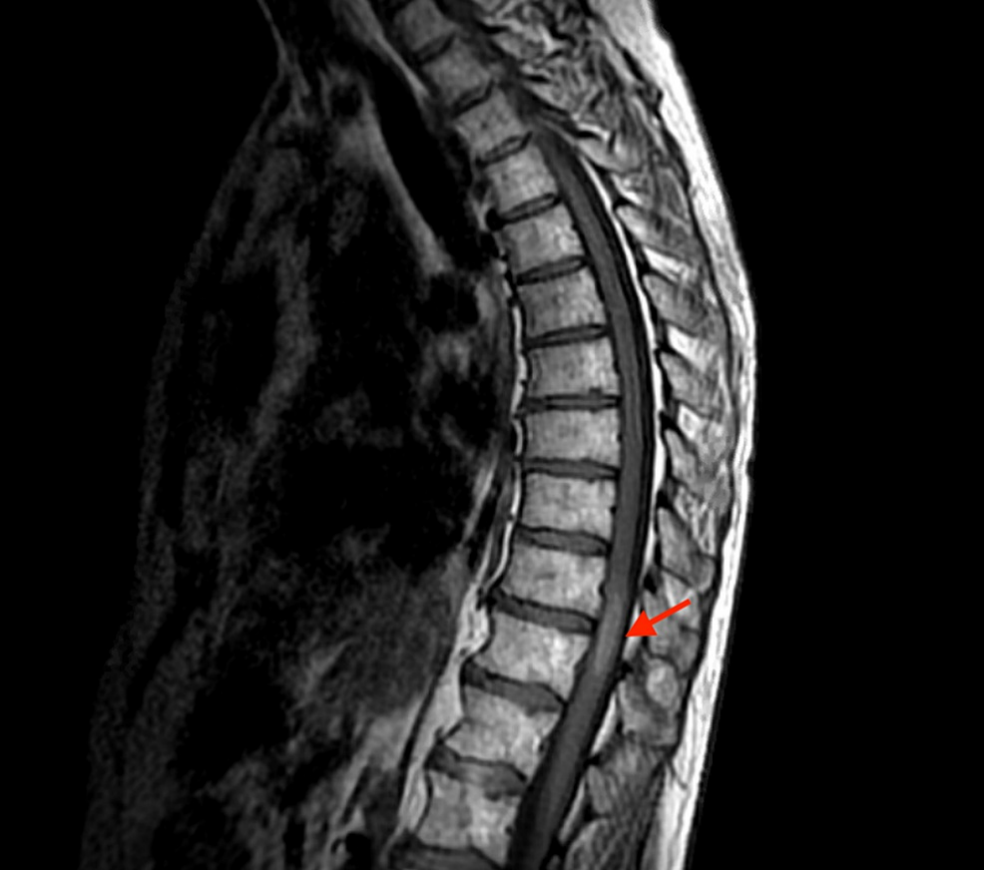
MRI of thoracic spine, T1 sagittal view. Minimal hyperintense T1 signal, suggesting intrinsic hemorrhage.

**Figure 2:**
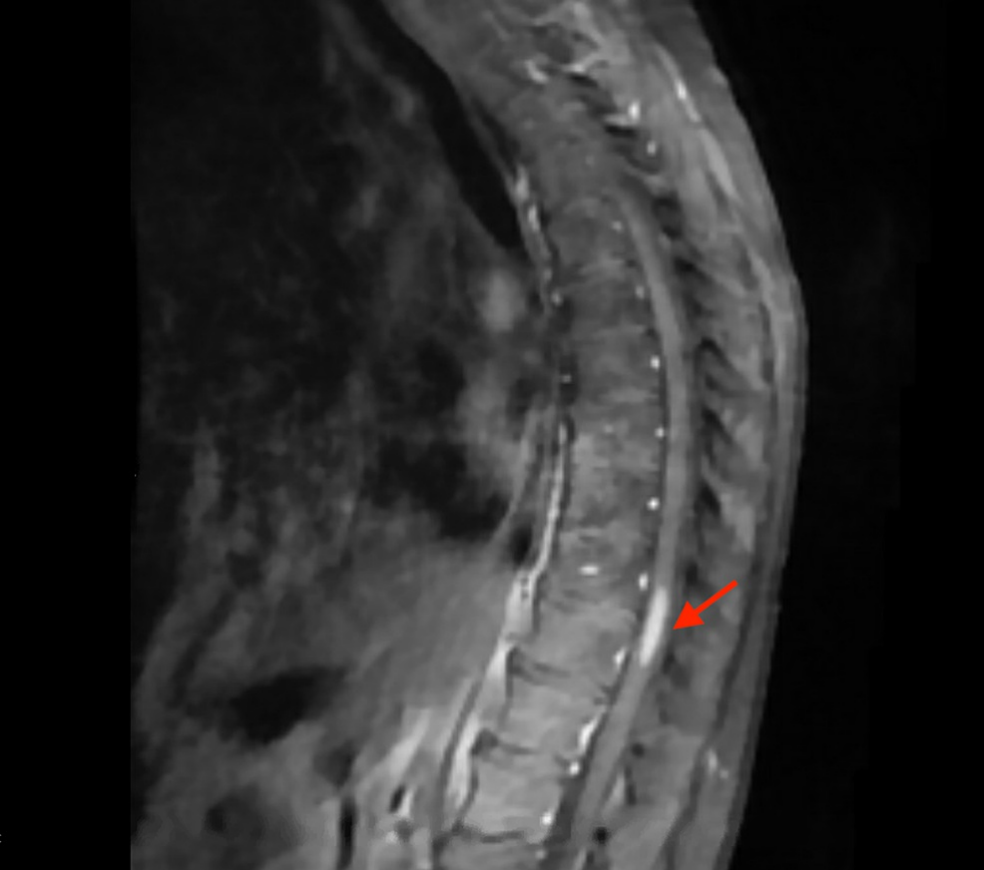
MRI of thoracic spine, T1 sagittal view with intravenous contrast and fat suppression. Moderate enhancement on intra-medullary lesion with intravenous contrast.

**Figure 3:**
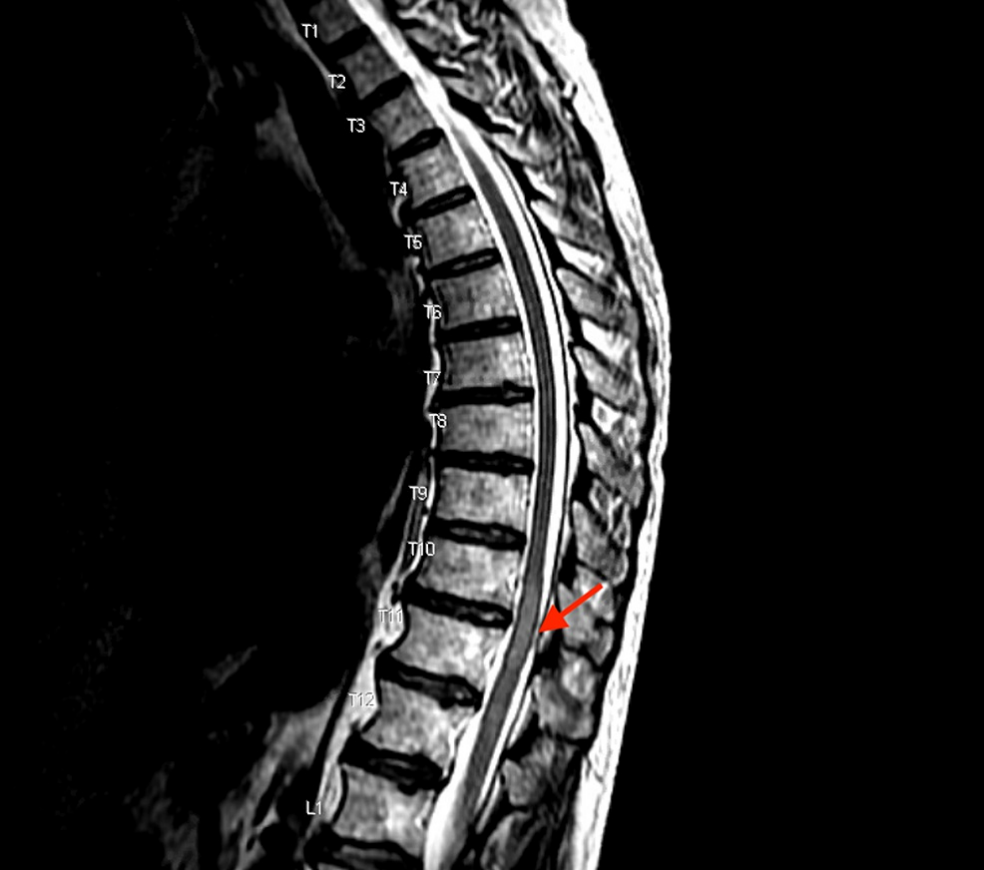
MRI of thoracic spine, T2 sagittal view. A slight hyperintense signal on T2 weighted sequence can be visualized.

Magnetic resonance imaging (MRI) studies of the cervical and lumbosacral spine revealed no abnormalities that could explain patient’s symptoms. MRI of thoracic spine with contrast demonstrated an expansile, intramedullary lesion in the thoracic cord at the levels T10 and T11. There was minimal hyperintense T1 signal, suggestive of intrinsic hemorrhage (Figure 1), and moderate enhancement with intravenous contrast (Figure 2). There was a slight increase in signal on T2 weighted sequences (Figure 3). This raised concern for a spinal cord malignancy like ependymoma, astrocytoma, hemangioblastoma, melanoma, or metastatic disease.

**Figure 4:**
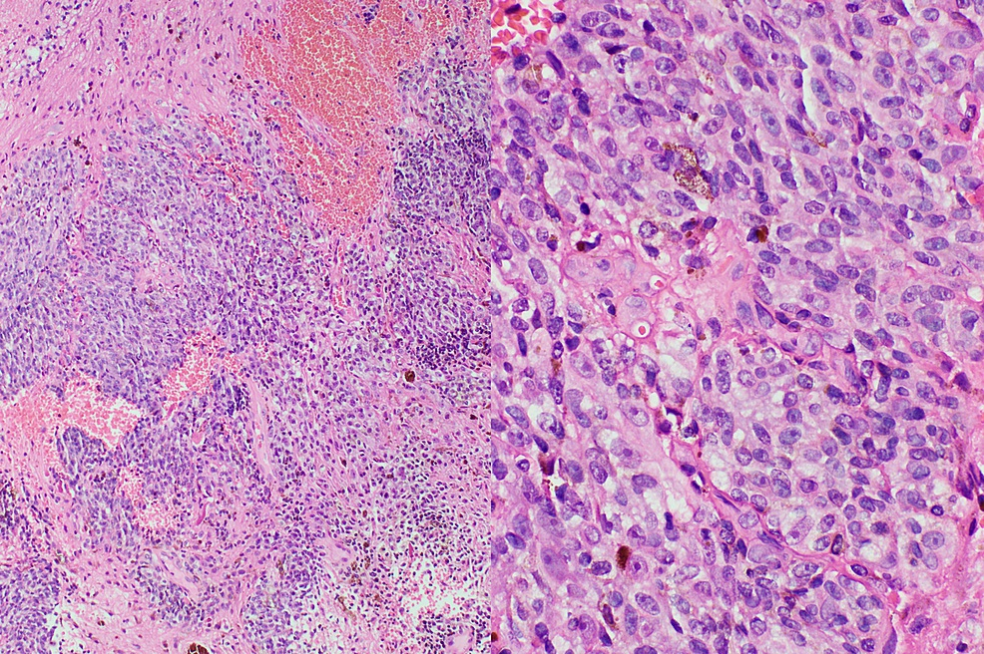
Low and high-power view of H&E stained sections. Melanoma in cellular nests composed of large cells with prominent nucleoli but low N-C ratio, with pigments present in some tumor cells. H&E: hematoxylin and eosin.

**Figure 5:**
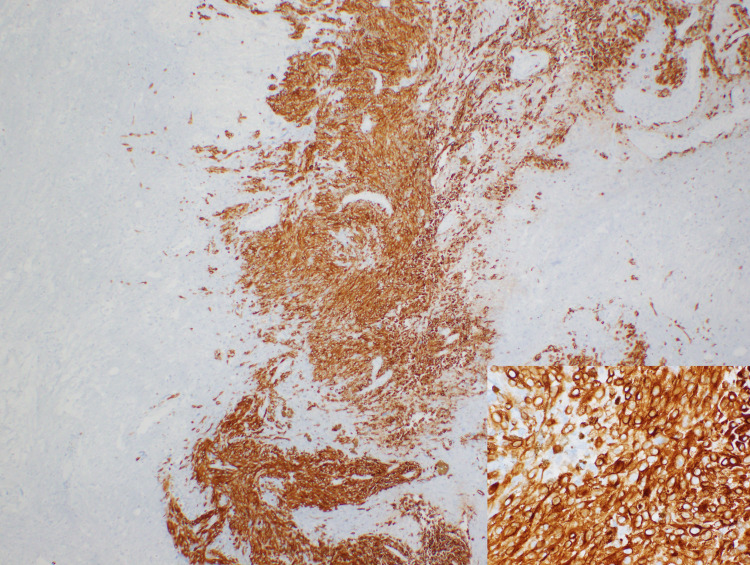
IHC stain for MART-1/Melan-A. IHC shows cytoplasmic staining in melanoma cells. IHC: immunohistochemistry.

**Figure 6:**
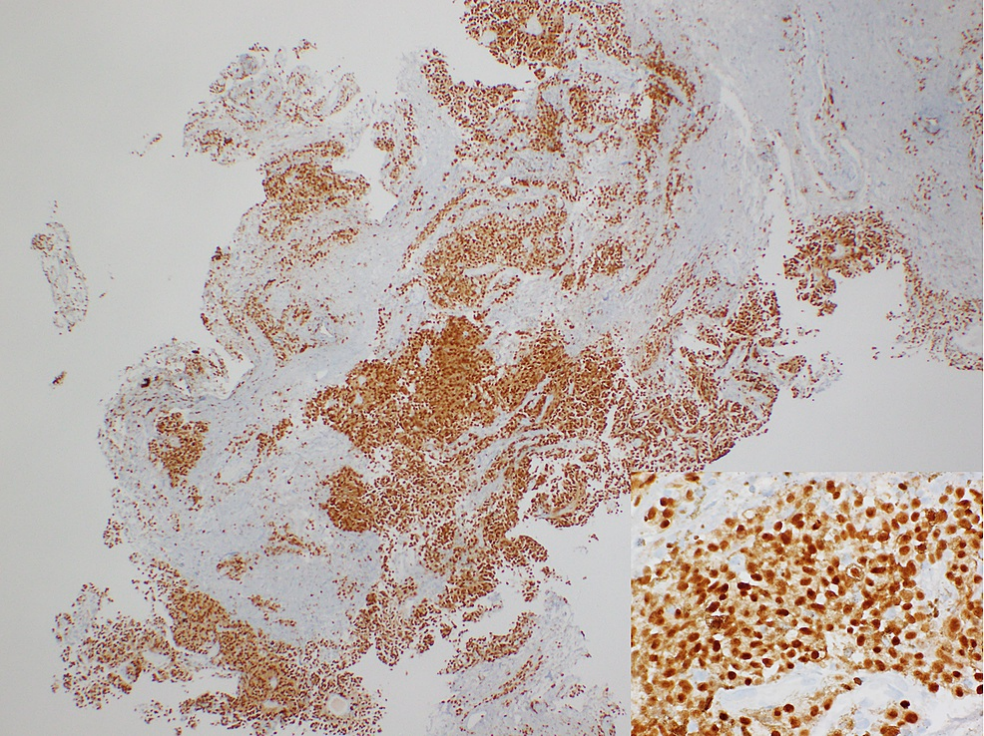
IHC stain for MITF. IHC shows nuclear staining in melanoma cells. MITF: microphthalmia-associated transcription factor; IHC: immunohistochemistry.

Patient underwent a T9-T11 laminectomy and resection of this T10-T11 intramedullary tumor using ultrasonographic spinal neuro-navigation, neurophysiological monitoring, and intradural operative microscope. Gross total resection (GTR) was achieved. A dark brown-green colored tumor was visualized and sent for intra-operative frozen section pathologic analysis which revealed an epithelioid neoplasm with marked pigment deposition. On histopathology, the tumor was demarcated from spinal cord tissue and was composed of nests of medium- to large-sized cells with prominent nucleoli, low N-C (nuclear to cytoplasmic) ratio, appreciable mitotic activity, and fine granular cytoplasmic melanin pigments (Figure 4). Immunohistochemically, the tumor was positive for S-100 protein, Melan-A or MART-1 (Figure 5) and MITF (Figure 6), confirming malignant melanoma. There was prominent lymphoplasmacytic reaction to the tumor, together with hemosiderin deposits indicating prior hemorrhage. Subsequent workup including a careful skin exam, ophthalmological evaluation, and whole-body PET-CT revealed no evidence of extra-spinal malignancy. Of note, the patient had a colonoscopy before GTR that had not shown any evidence of melanoma in colonic mucosa. However, an upper gastrointestinal endoscopy was deferred in this case due to the high risk of complications after spinal surgery. Given the above workup, the patient was diagnosed with Primary intramedullary malignant melanoma of thoracic spine.

**Figure 7:**
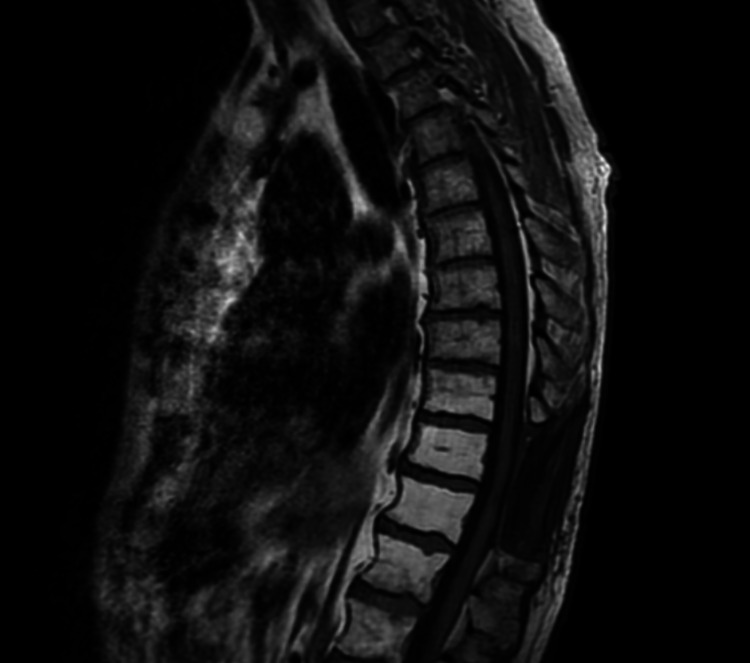
MRI of thoracic spine, T1 sagittal view. No evidence of disease recurrence.

**Figure 8:**
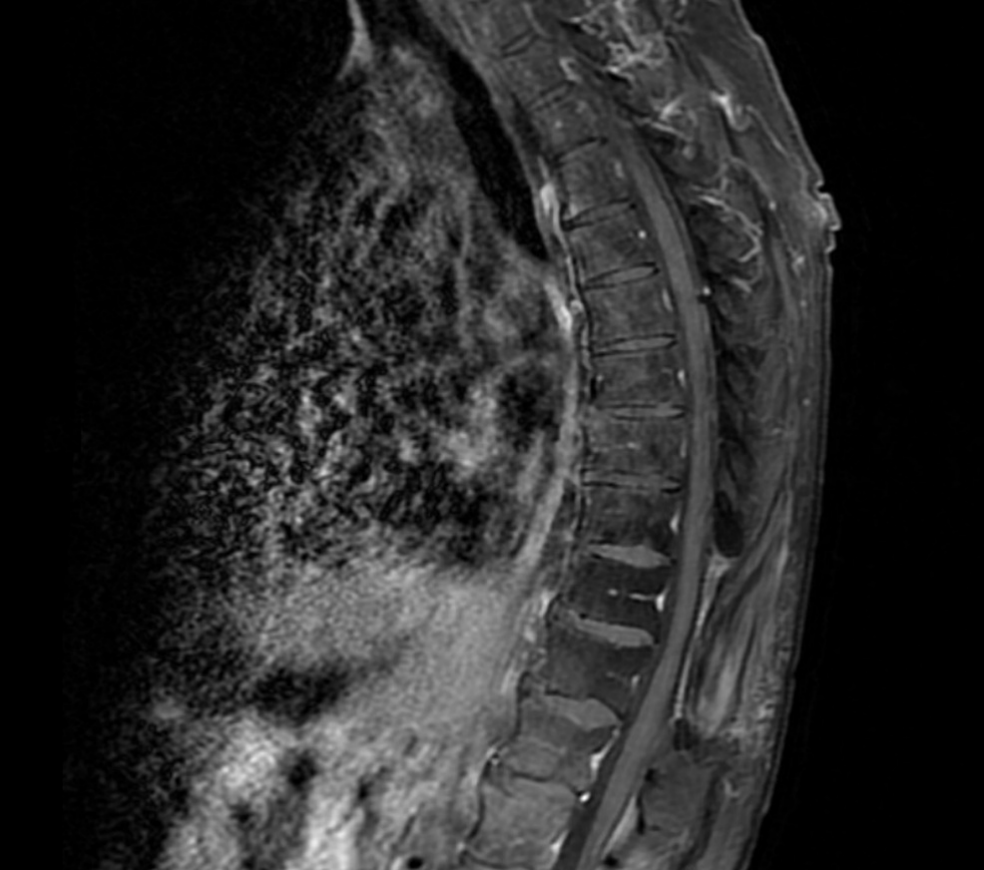
MRI of thoracic spine, T1 sagittal view with intravenous contrast and fat suppression No evidence of disease recurrence.

**Figure 9:**
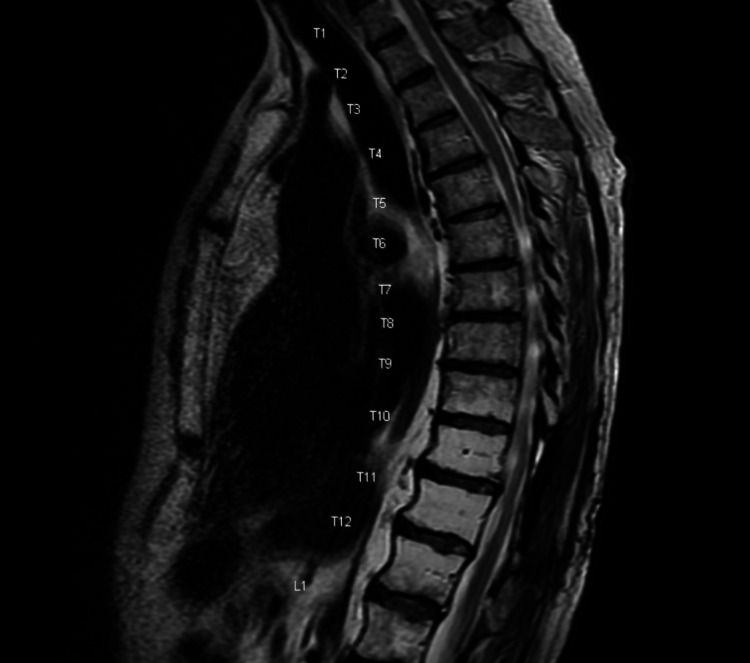
MRI of thoracic spine, T2 sagittal view. No evidence of disease recurrence.

After GTR, the patient received adjuvant radiation therapy to the lower thoracic spine surgical bed and completed a total dose of 5040cGy in twenty-eight fractions. Adjuvant checkpoint inhibitor therapy with nivolumab was subsequently administered at 480 mg intravenously every four weeks. The patient tolerated eleven of thirteen cycles of nivolumab after which it was discontinued when he developed grade III pneumonitis (immune-related adverse effect) that was effectively treated with high dose steroid therapy that was tapered over several weeks. Fifteen months after initial spinal surgery patient’s lower extremity weakness, hypesthesia, and sphincter dysfunction has nearly resolved. Most recent surveillance MRI scan performed 12 months after spinal surgery showed no evidence of disease recurrence (Figures 7, 8, 9). The patient continues to follow-up regularly with Oncology.

 

## Discussion

Primary CNS melanoma is a malignant tumor that was first described by Hirschberg in 1906 [4]. This rare entity accounts for only 1% of all melanomas and has an estimated incidence of 0.005 cases per 100,000. Oncogenesis is hypothesized to occur from neural crest cells that fail to migrate during embryogenesis and reside in the neural tube. Later in life, dysregulated signaling between these neural crest cells and adjacent cells causes disruption in normal differentiation and maturation of these cells. It has also been hypothesized that primary CNS melanoma in atypical sites may stem from melanocyte precursors that accompany the pial sheaths of vascular bundles. Oncogenesis from neuroectodermal cells with abnormalities in cellular migration has also been suggested [5,6]. Primary spinal melanoma(PSM) is even more unique and most of its cases seem to arise in the thoracic spinal cord [7]. Location of these tumors can be intra- or extramedullary, leptomeningeal, or extradural. Intramedullary location of the neoplasm, as seen in our case, is very rare [1]. Few population studies on primary CNS melanomas published to date show a similar predisposition of males and females to be affected with peak incidence reported in the fifth decade of life [2,8]. Symptoms of PSM depend on exact location of the tumor and are usually sub-acute or insidious in presentation, except in case of hemorrhage which may cause acute worsening of neurological symptoms. Patients may present with neck or back pain, signs of progressive asymmetric sensory and motor myelopathy, or sphincter dysfunction [9].

Factors to be considered in diagnosis of primary CNS melanoma were first described by Hayward in 1976: absence of other CNS tumors, lack of extra-CNS lesions, and pathological confirmation [3]. The diagnostic approach to primary spinal melanoma is based on excluding primary site of origin outside the spinal cord for which a thorough ophthalmologic, dermatologic, and gastrointestinal examination is required [10]. In our patient, a comprehensive eye exam, skin inspection, and colonoscopy did not reveal an extra-spinal primary leading to the diagnosis of primary spinal melanoma. MRI is the imaging modality of choice for diagnosis but there are no distinct MRI findings that can differentiate primary spinal melanoma from other pigmented tumors of the spinal cord such as extra-spinal metastatic melanoma, and hemorrhagic neoplasms, like ependymoma and astrocytoma. The imaging features of primary spinal melanoma can vary depending on the extent of melanocytic content and based on the absence or presence of hemorrhage. Most lesions show hyperintense signals on T1-weighted images while the T2-weighted images may demonstrate iso or hypointense signals compared to normal cord. Homogeneous pattern of enhancement on gadolinium post-contrast images has often been reported but sometimes the pattern can be peripheral, inhomogeneous, or nodular [1,6,11]. Malignant melanomas have high metabolic activity and function and a PET/CT is helpful for the evaluation of local versus metastatic spread from distant disease [12]. None of these imaging modalities are highly specific, therefore a definitive diagnosis of PSM is made on pathologic studies. Hyperplastic sheets of spindled or epithelioid cells can be seen and may show significant pleomorphism. Most tumors have some degree of cytoplasmic melanin but rare instances of amelanotic melanoma have been reported. Other features like prominent nucleoli, atypical mitoses, tissue invasion, and necrosis can be seen as well [6,13]. Key immunohistochemical markers for diagnosis include human melanoma black-45(HMB-45): a marker of melanocytic differentiation and melanosome formation, S-100 protein, Melan-A: melanocytic lineage-specific marker, and microphthalmia-associated transcription factor(MITF): a major transcriptional regulator of the melanocytic cell lineage [13-15].

In our patient, minimal T1 hyperintensity and moderate enhancement after intravenous contrast was characteristic of malignant melanoma. PET/CT scan demonstrated a primary spinal cord lesion and pathologic findings consolidated the diagnosis of primary malignant melanoma of the spine.

Because, PSM is a rare tumor, there are no definitive guidelines for its treatment. Overwhelming consensus based on published literature favors gross total resection (GTR) as cornerstone of treatment. GTR yields improved survival and mortality outcomes. Adjuvant radiation treatment (RT) with GTR may decrease odds of local recurrence compared to GTR alone but Zhang et al. did not observe any added survival benefit [2]. Puyana et al. concluded that compared to GTR, sub-total resection (STR) does not offer a survival benefit without addition of adjuvant radiation therapy (RT) and hence in such cases where a complete surgical excision or GTR is not possible, adjuvant radiation becomes a more vital element of treatment, akin to the treatment of resected cutaneous melanomas with positive margins without further surgery being feasible [8]. Surgical excision either GTR or STR improves overall survival compared to biopsy alone. The role of adjuvant systemic therapy remains less clear. However, adjuvant systemic therapy is generally administered in combination with surgical excision and adjuvant radiation. Case studies have shown variable survival outcomes with use of adjuvant dacarbazine or temozolomide [16]. Improved disease control and survival with the use of high dose systemic interferon (INF)-beta or INF-alpha has also been described in few cases [17]. Novel biologics and targeted therapies are promising and have a favorable side effect profile than cytotoxic chemotherapies. Targeted therapy agents like dabrafenib and vemurafenib can be effective adjuvant agents in patients with BRAF mutant melanomas [18]. Immune checkpoint inhibitor therapy with cytotoxic T lymphocyte antigen-4 inhibitor Ipilimumab and programmed death-1 inhibitors Nivolumab and Pembrolizumab has shown positive outcomes in patients with advanced melanomas [19]. Efficacy in primary spinal melanoma has not been established. Oncogenic mutations in ROS-1, ALK, NRAS, P13K/AKT/mTOR pathway, and GNAQ and GNA11 genes are also being investigated for therapeutic potential with targeted agents [20]. In the case of our patient, complete surgical resection, adjuvant radiation therapy and adjuvant immunotherapy with Nivolumab has shown a promising outcome in the form of recurrence-free survival over 15 months since surgery, suggesting a triple modality approach may be an effective option for the management of patients with this rare disease.

## Conclusions

Primary intramedullary spinal melanoma is extremely rare and often arises from thoracic spinal cord. Characteristic MRI findings can indicate spinal melanoma, but a definitive diagnosis of primary spinal melanoma needs pathologic confirmation and ruling out extra-spinal melanoma. GTR should always be attempted to yield most promising survival and mortality outcomes. Adjuvant radiation limits local recurrence but the role of adjuvant chemotherapy, targeted therapy, and immunotherapy to decrease the risk of developing distant disease needs to be explored further. Our case provides a unique insight into benefits of adjuvant immunotherapy with Nivolumab in addition to GTR and RT, and highlights a shifting paradigm in CNS melanoma treatment options.
